# Access to tuberculosis care in South Africa during the COVID-19 pandemic: A scoping review

**DOI:** 10.4102/phcfm.v17i1.4944

**Published:** 2025-07-30

**Authors:** Kayla Appel, Faeez Nackerdien, Carmen S. Christian

**Affiliations:** 1 Department of Economics, Faculty of Economics and Management Sciences, University of the Western Cape, Bellville, South Africa

**Keywords:** tuberculosis, pandemic, COVID-19, healthcare access, South Africa, Penchansky and Thomas framework, demand-side issues, supply-side issues

## Abstract

**Background:**

Tuberculosis (TB) remains a major public health issue in South Africa, a high-burden TB country. The coronavirus disease 2019 (COVID-19) pandemic has exacerbated challenges in accessing essential TB services. This scoping review explores how access to TB care was impacted during the pandemic.

**Aim:**

This research aimed to review original studies on access to TB care in South Africa during the COVID-19 pandemic using a scoping review methodology.

**Method:**

A scoping review was conducted using the Preferred Reporting Items for Systematic Reviews and Meta-Analyses for Scoping Reviews (PRISMA-ScR) guidelines. Five databases were systematically searched for original peer-reviewed research published between 2020 and 2022. Data were extracted and synthesised using the Penchansky and Thomas framework of healthcare access.

**Results:**

Three studies met the inclusion criteria. The review identified significant disruptions in TB service delivery during the pandemic, including reduced diagnostic capacity, healthcare facility closures and economic barriers. Patients reported delayed diagnoses and increased stigma, while healthcare workers faced resource shortages and operational challenges.

**Conclusion:**

The COVID-19 pandemic has exacerbated pre-existing barriers to TB care in South Africa, highlighting critical gaps in healthcare delivery. This review provides insights into the challenges faced and emphasises the need for resilient health systems to sustain TB care during future health crises.

**Contribution:**

This article highlights the impact of the COVID-19 pandemic on TB care access in South Africa, identifying key barriers across healthcare access dimensions and offering recommendations to improve TB care delivery during public health emergencies.

## Introduction

In South Africa, one of 30 countries with a high burden of tuberculosis (TB), TB remains a global pandemic and a public health crisis.^1^ Despite slight improvements in TB outcomes over the past decade, tuberculosis remains one of the primary causes of morbidity in South Africa. Furthermore, TB management has historically been overshadowed by the highly prioritised infectious human immunodeficiency virus (HIV) in terms of research and development, even though 60% of TB patients in South Africa are co-infected with both TB and HIV.^2^

More recently, TB has yet again taken a back seat to another infectious disease: the coronavirus disease 2019 (COVID-19) pandemic. The World Health Organization’s (WHO’s) latest global TB report has warned that in many countries, the pandemic has reversed years of incremental progress made in providing essential TB services and reducing the TB disease burden.^3^ South Africa is no exception to this regression; the rate at which the TB incidence decreased in South Africa from 2015 to 2019 has slowed down since the pandemic and was coupled with an increase in TB mortality.^3^

The unprecedented crisis that the COVID-19 pandemic presented to South Africa’s health system has underscored the need to strengthen resilience in the health system, particularly in its approach to managing TB. However, not enough research has been generated yet to provide a deep enough understanding of how access to TB care was affected during the pandemic, creating a pressing need to investigate this black box and explore how best to move forward with original research. Investigating these disruptions is essential for aligning with South Africa’s National Strategic Plan for HIV, TB and Sexually Transmitted Infections (STIs) (2023–2028) and the WHO’s End TB Strategy, which aims to reduce TB cases by 90% and deaths by 95% by 2035.^4,5^ This study will provide insights to guide policy and inform future research on TB management in the context of the COVID-19 pandemic. By systematically identifying these gaps, the study will help ensure that subsequent research can effectively contribute to societal transformation and development in South Africa and other developing countries.^6^

According to Penchansky and Thomas, access is a notion that reflects the level of ‘fit’ that exists between the system and its clients.^7^ In essence, if a client (e.g. patient) has a set of needs (based on their characteristics and expectations), it refers to how adequately the system (e.g. health system) can meet the client’s needs. Access is seen as a broad term that encapsulates a number of more focused areas where the patient and the health system can work together.^7^ Penchansky’s framework defines access across five dimensions:

- Availability: The correlation between the quantity and kind of current services (and resources) and the quantity and nature of client needs.
- Accessibility: The relationship between a client’s location and the supplier location, accounting for the client’s transportation resources as well as trip expenses, time and distance.
- Accommodation: The connection between how supply resources are set up to accommodate customers (such as appointment scheduling, operating hours, walk-in facilities and phone services) and how well-suited the customers feel these elements are.
- Affordability: How service costs and insurance or deposit requirements relate to a client’s income, financial capacity and current health insurance.
- Acceptability: The association between clients’ perceptions of acceptable personal traits of clients and their perceptions of the practice and personal traits of current providers is referred to as acceptability.

This research aimed to review original studies on access to TB care in South Africa during the COVID-19 pandemic using a scoping review method. The objectives of this scoping review were to:

- Describe demand-side issues with access to TB care during the COVID-19 pandemic
- Describe supply-side issues with access to TB care during the COVID-19 pandemic
- Compare access to TB care before and during the COVID-19 pandemic.

## Methods

### Study design

We followed the Preferred Reporting Items for Systematic Reviews and Meta-Analyses for Scoping Reviews (PRISMA-ScR) guidelines when conducting this scoping review. The search and selection strategies were implemented by three researchers who replicated and audited each other’s work. Cases of conflicting study selections were discussed by the research team, and the outcome was determined through joint consensus.

A standardised template was designed in Microsoft Excel and used to capture the searches, collect the relevant extracted data and compare study characteristics. We used a narrative approach to integrate and synthesise the findings according to Penchansky and Thomas’s access framework, after which knowledge gaps were identified.

### Search strategy

We systematically searched five databases (Google Scholar, PubMed, SocIndex, Cumulative Index to Nursing and Allied Health Literature [CINAHL] and Academic Search) to identify studies from 2020 to 2022 using the following search strings: (1) ‘tuberculosis’ AND ‘access’ AND ‘COVID-19’ AND ‘South Africa’; (2) ‘tuberculosis’ AND ‘servic*’ AND ‘access’ AND ‘COVID-19’ AND ‘South Africa’ as indicated in Box 1. The literature search was conducted from 21 September 2022 to 05 October 2022.

BOX 1 Search strings used in scoping review.

**Search strings:**

- “tuberculosis” AND “access” AND “COVID-19” AND “South Africa”
- “tuberculosis” AND “servic*” AND “access” AND “COVID-19” AND “South Africa”

The search rule was implemented to search for articles within the databases that met the inclusion criteria of this review. The articles were sorted by relevance and limited to 10 per page. The search process continued until the last three articles were irrelevant (after 13 pages, in this case). This search was completed on 05 October 2022, and all duplicate articles were removed by 06 March 2023.

### Selection criteria

The inclusion criteria were specified as original research published in peer-reviewed journals that contained findings directly linked to our objectives (i.e. it explained demand-side and supply-side issues related to access to TB care during the COVID-19 pandemic). Only studies written in English and that had full text available were selected.

The exclusion parameters related to the study type included thoughts, critiques, editorials, comments and opinions. Reviews (i.e. scoping, systematic, narrative and literature reviews) were also excluded from our final sample. However, we retained reviews (i.e. scoping, systematic, narrative and literature reviews) in the initial rounds of the searches until the penultimate step in the search and selection process. These reviews were then scanned for additional original research that was not found in the first rounds of searches using the search strings. Once this step was completed, the reviews were excluded, and the additional original research sources were selected and added to the screening round. Online Appendix 1 shows the exclusion process of all the articles by original reviews.

All selected studies were screened by their titles and abstracts and balanced with inclusion and exclusion criteria. Studies that made it through this round of screening were downloaded, and the full texts were further read by the researchers. Thereafter, the researchers reached consensus on which studies would be included in the final sample for review.

### Extraction of data

Data were extracted into a standardised Microsoft Excel template according to the following fields:

1. Author.
2. Year of publication.
3. Location of the study population (in South Africa).
4. Aim or purpose of the publication.
5. Methods.
6. Demand-side issues with access to TB care during the COVID-19 pandemic.
7. Supply-side issues with access to TB care during the COVID-19 pandemic.
8. Demand-side issues with access to TB care before the COVID-19 pandemic.
9. Supply-side issues with access to TB care before the COVID-19 pandemic.
10. Study limitations as reported by the authors.

Data for extraction points (1) to (5) are presented in Table 1, while data for extraction points (1) to (3) and (6) to (10) are presented in Table 2.

**TABLE 1:** Three included studies in the scoping review.

Article	First author name	Year of publication	Location	Aim or purpose	Methods
(9)	Addo et al.	2022	South Africa, all provinces	To understand the common perspectives of TB patients across Brazil, Russia, India, China and South Africa throughout their disease journey, including the emotional, psychological and practical challenges that patients and their families face.	A qualitative market research study was conducted between July 2020 and February 2021. A total of 40 TB patients (8 from each country) completed health questionnaires, video/telephone interviews and diaries regarding their experiences of TB, with an additional 52 household members being interviewed. Patients were sought at different stages of their TB treatment journey, from a variety of socio-economic groups, with or without TB risk factors. Anonymised data underwent triangulation and thematic analysis by iterative coding of statements.
(10)	Pillay et al.	2021	South Africa, all provinces	To analyse the effects of COVID-19 on normal health services in South Africa, as well as the constraints imposed to limit viral transmission.	Data were regularly collected using the District Health Information System (DHIS) from 2019 to 2020.
(11)	Watkins et al.	2021	Gauteng Province, South Africa	To understand how trust plays out in the workplace of community health workers in South Africa.	Conducted during the observation phase of a 3-year intervention study, interviews, focus groups and observations were conducted with patients, community health workers, their supervisors and facility managers in Sedibeng.

Note: Please see the full reference list of the article, Appel K, Nackerdien F, Christian CS. Access to tuberculosis care in South Africa during the COVID-19 pandemic: A scoping review. Afr J Prm Health Care Fam Med. 2025;17(1), a4944. https://doi.org/10.4102/phcfm.v17i1.4944

TB, tuberculosis; COVID-19, coronavirus disease 2019.

**TABLE 2:** Descriptive summary of included studies.

Article	First author name	Demand-side issues with access to TB care during the COVID-19 pandemic	Supply-side issues with access to TB care during the COVID-19 pandemic	Demand-side issues with access to TB care before the COVID-19 pandemic	Supply-side issues with access to TB care before the COVID-19 pandemic	Study limitations as reported by the authors
(9)	Addo et al.	TB patients’ fear of COVID-19 led to worsening conditions and transmission to family members. Restrictions on movement and hospital admissions further limited access to care. Stigma associated with both COVID-19 and TB led to isolation and reduced social interactions.	Drug shortages and COVID-19-related disruptions to TB services during the pandemic raised concerns about treatment disruptions and multidrug-resistant TB. Access to care was made worse by overcrowded medical facilities and limitations on non-COVID admissions, which created challenges for patients.	Stigma related to TB existed before the pandemic, and patients faced isolation from social networks. In some regions, TB was associated with poverty and marginalised groups, further alienating patients.	Patients face challenges in accessing TB medications because of long queues, poor public healthcare service, drug stockouts and reliance on private healthcare for better treatment.	Did not use a random sample, analyse treatment suitability or compare experiences with drug-susceptible vs multidrug-resistant TB. Patient pathways were not fully integrated into the thematic analysis, and UK-based analysts may have lacked cultural understanding of some patient experiences.
(10)	Pillay et al.	The fear of COVID-19 in healthcare facilities and restrictions on movement during lockdown discouraged patients from seeking care, leading to a reduction in visits to health centres. Transportation issues and closures of facilities further limited patients’ access to healthcare services, particularly for TB care.	Health facilities experienced staffing shortages because of COVID-19 infection, isolation and redeployment, reducing routine services, including TB care. GeneXpert tests for TB decreased by 26% between 2019 and 2020, and the number of people diagnosed with TB also declined during the pandemic.	Not applicable.	Not applicable.	The use of 2019 as a baseline, its appraisal of the data’s quality, its dependence on a single data source, and its variable data quality and lack of explicit attributions.
(11)	Watkins et al.	Not applicable.	Not applicable.	Because poverty, immigrant status and societal stigma and discrimination make it difficult for patients to receive regular care, they frequently put off seeking care. Injustices in the healthcare system are made worse by the fact that migrants frequently lack documentation and a stable income.	Lack of resources, structural problems including community poverty, and a lack of identification among migrant communities made it difficult for CHWs to deliver safe and effective care. Patient privacy and infection control were hampered by physical problems in medical institutions.	Participants’ opinions on trust were limited because the data was not particularly collected for trust study.

Note: Please see the full reference list of the article, Appel K, Nackerdien F, Christian CS. Access to tuberculosis care in South Africa during the COVID-19 pandemic: A scoping review. Afr J Prm Health Care Fam Med. 2025;17(1), a4944. https://doi.org/10.4102/phcfm.v17i1.4944

TB, tuberculosis; COVID-19, coronavirus disease 2019.

## Review findings

**FIGURE 1:**
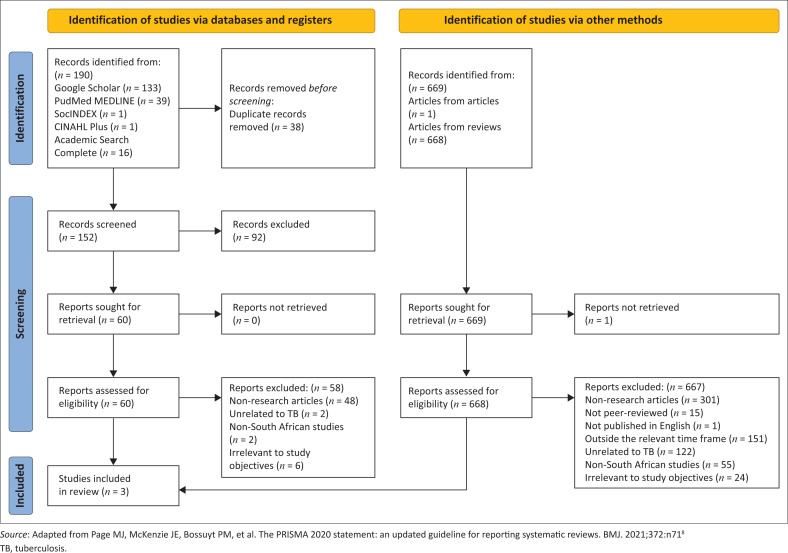
PRISMA flow diagram for selection of studies.

The flow of our sample collection is described following the PRISMA flow diagram in Figure 1. For the process of identifying studies via databases, 190 studies were initially identified from five database sources. After the removal of 38 duplicate records, 152 unique records were screened, whereby 60 reports were sought for full-text retrieval and eligibility assessment. Out of these, 58 reports were eliminated.

A total of 669 records were collected for the identification of research using alternative methods; 668 records were from reviews that were not included in the prior process, and 1 record came from papers cited in other studies. After screening, 669 reports were sought for full-text retrieval, and only 1 report could not be retrieved. Of the 668 reports assessed for eligibility, 667 were excluded.

The paucity of original studies that met the inclusion criteria and study aim resulted in a small sample size. Most of the information in the review articles came from news items and reports. These sources did not meet our criteria for original research and were therefore excluded. As a result, the final sample did not include any references used in the reviews. In the end, three papers from these database searches and articles from sources were included in the review.

### Features of included articles

Table 1 provides an overview of the methodological characteristics of the three included articles conducted in South Africa, arranged by the first author’s name. Two of the three articles were published in 2021, and one article was published in 2022. Two of the articles employed a qualitative approach design, and one article used a quantitative approach.

The first article reported on the emotional and psychological challenges of TB patients throughout their treatment journey and also the challenges patients and their families faced across the BRICS nations. The second article analysed the effects as well as the constraints of COVID-19 on health services in South Africa from 2019 to 2020 through data collected from the District Health Information System (DHIS). The last article aimed to understand the role of trust in the workplace of community health workers (CHWs). Together these articles highlighted the various challenges TB patients faced during treatment, how COVID-19 strained the delivery of healthcare services, and the challenges CHWs faced in providing treatment during the pandemic.

Table 2 summarises the demand-side and supply-side issues with access to TB care before and during the COVID-19 pandemic, as well as the study limitations of the three included studies. On the demand side, TB patients had been discouraged from seeking treatment not only by lockdowns and fear of infection but also by the social stigma of COVID-19, including blame, social exclusion and being labelled contagious, which made them avoid clinics. These factors delayed diagnosis and worsened treatment outcomes. Before the pandemic, marginalised groups were disproportionately affected by problems like poverty, stigma and long wait times.^9,11^

On the supply side, inadequate funding and overcrowded facilities were two instances of structural issues that hindered CHWs’ ability to deliver quality care. They also struggled because of a lack of resources, poverty and difficulty identifying migrants. Drug shortages and COVID-19-related disruptions raised concerns about treatment interruptions and multidrug-resistant TB. Between 2019 and 2020, GeneXpert TB tests decreased by 26%, and during the pandemic, fewer TB diagnoses were made because of staff shortages and reduced routine services. Poor infrastructure, long queues, drug shortages and reliance on private healthcare further restricted access to TB medications. Among the limitations noted by the authors of the three studies were the use of non-random samples, secondary data and a lack of attention to certain subjects such as trust.

## Discussion

This section provides a comprehensive discussion of the key findings from the scoping review, categorised into five dimensions of healthcare access: availability, accessibility, accommodation, affordability and acceptability. Each dimension highlights the impact of the COVID-19 pandemic on TB care in South Africa.

### Availability

Availability refers to the presence and adequacy of health services and resources, such as sufficient health facilities and infrastructure, adequate staffing, diagnostic tools and medication to meet patient needs. Before the COVID-19 pandemic, South Africa’s TB care system faced ongoing issues with drug shortages and inconsistent supply chains, particularly in rural areas^9,11^ These issues prevented patients from receiving treatment, raising the risk of drug-resistant tuberculosis. Despite efforts to integrate tuberculosis services into general healthcare, resources remained insufficient.^12^

The pandemic further amplified these issues by reallocating resources to COVID-19 response operations. GeneXpert TB tests, which are vital for early TB identification, declined by 26% between 2019 and 2020.^10^ This reduction is noteworthy since early detection is critical for reducing TB transmission.^13^ The reallocation of healthcare resources not only slowed TB diagnosis but also reduced patient monitoring and interrupted follow-up care, raising the risk of further transmission.^9,10^

The findings from South Africa are consistent with global trends reported by the WHO, which found a 15% decline in enrolment in drug-resistant tuberculosis therapy worldwide in 2020, with only minimal recovery by 2021.^14^ Furthermore, WHO predicted that pandemic-related disruptions would result in 4.7 million new TB cases and 1 million TB fatalities by 2025,^15^ further highlighting the far-reaching consequences of the pandemic’s restricted availability of TB treatment.

Healthcare professionals reported that their workloads had increased and that they had limited access to personal protective equipment, which added to the strain on service delivery.^10^ The increasing workload, along with inadequate personal protective equipment, raised the risk of infection among healthcare professionals, restricting their ability to offer care and further highlighting the critical requirement for robust health systems that can provide normal tuberculosis care amid public health emergencies.

### Accessibility

Accessibility focuses on the geographical location of healthcare services and whether they are within reasonable proximity to patients with regard to time and distance for patients to access these services. South Africa faced severe accessibility issues even before the pandemic, particularly for patients who resided in rural and underdeveloped areas of the country and frequently had to travel long distances to reach medical facilities. Long waiting times and poor transportation infrastructure exacerbated these issues.^9^

The COVID-19 pandemic exacerbated these issues by imposing strict lockdown measures and movement restrictions, which significantly limited patients’ ability to access care. Restrictions on movement, the curtailment of public transportation and fear of exposure to COVID-19 in healthcare settings further discouraged many patients from visiting medical facilities. A national survey found that 57% of individuals were apprehensive about visiting clinics or hospitals during the lockdown, primarily because of concerns about being exposed to COVID-19.^11^ As a result, many patients missed crucial medical appointments, delayed treatments and experienced interruptions in their care.^9,10^

Additionally, health facilities experienced a decline in the number of TB and HIV patients collecting their medication on schedule, further highlighting the impact of accessibility barriers during the lockdown.^10,11^ In one province alone, over 1000 TB patients and nearly 11 000 HIV patients did not collect their medications on time.^16^ Additionally, long waiting times and limited services in public healthcare systems, already problematic before the pandemic, were worsened by resource redirection toward the COVID-19 response, which further diminished patients’ ability to access necessary care during the pandemic.^9^

### Accommodation

Accommodation refers to how healthcare facilities are organised to meet patients’ needs, including their operating hours, how appointments are scheduled and whether they can handle patients’ time constraints and preferences. Even before the pandemic, South Africa’s healthcare system was already struggling with long waiting times, limited facilities and work overload, which made it difficult for TB patients to get the care they needed on time.^9^ These challenges caused delays in starting treatment, disrupted patient monitoring and led to poor follow-up, which likely worsened the spread of TB and patient outcomes.

During the COVID-19 pandemic, the situation worsened as healthcare facilities prioritised COVID-19 cases, significantly reducing their capacity to accommodate TB patients. Non-COVID-related admissions, including those for TB treatment, could no longer be accommodated in healthcare institutions because of the overwhelming number of COVID-19 cases at the time. Hospital admissions were limited because non-COVID cases were deprioritised.^9,10^

The poor implementation of TB infection control procedures in primary health care institutions made it even more difficult for healthcare providers to accommodate TB patients. Contradicting standards, such as the National Core Standards and the Ideal Clinic Policy, perplexed healthcare personnel, particularly regarding whether TB patients should be separated from other patients.^17^ Facility administrators stated that the inconsistent requirements made it impossible to maintain consistent infection control methods, resulting in service disruptions. They also stated that, despite being required to train their employees on TB infection management, they did not feel prepared to do so.^17^

The lack of clear direction and sufficient support hampered healthcare facilities’ capacity to address patients’ requirements. Healthcare professionals also faced additional obstacles, such as a lack of frequent health examinations and insufficient occupational health support.^9^ These gaps left many people feeling hopeless and unmotivated and unfavourable attitudes increased as long-standing issues, such as resource constraints and inadequate district-level support, went unsolved. Together, these challenges made it impossible for healthcare facilities to provide regular and timely tuberculosis treatment.

Community health workers (CHWs), who played a vital role in helping TB patients, faced additional challenges during the pandemic. Poor infrastructure, a shortage of necessary supplies and a lack of personal protective equipment made it difficult for CHWs to continue providing care.^9,10^ There were also persistent operational difficulties at healthcare institutions, with CHWs having to work in unfavourable environments with weak infrastructure.^11^

### Affordability

Affordability looks at whether patients can afford the associated expenditures of healthcare services without experiencing financial hardship. In this scoping review, only one article reported on affordability issues. This article mentioned that even though most healthcare systems supplied free TB medications to patients during the COVID-19 pandemic, they still faced financial difficulty as a result of lost wages and the increase in medical and travel expenses. Many patients reported being unable to afford transportation to healthcare facilities or private healthcare services when public facilities were inaccessible.^9^ Particularly in private healthcare, several patients struggled to pay for further tests or prescriptions out-of-pocket.^16^ Before the pandemic, affordability was already a barrier for many TB patients, particularly for those from low-income households who struggled with transport and supplementary costs. Many families were already living in poverty, and the financial hardships brought on by COVID-19 regulations further deepened their struggles, limiting their ability to access healthcare.^9^ The WHO established a task force in 2015 to create a generic protocol for TB patient cost surveys, which was published in 2017. The first national patient cost study conducted in South Africa during the COVID-19 pandemic found that family and patient earnings were already poor before TB diagnosis and considerably worsened following the diagnosis.^14^ Household income declined by 15.6% and individual income fell by 44.9%, emphasising the necessity of securing these revenues to avoid increased economic strain.^18^

These difficulties were worsened by the government’s limited social aid.^19^ For example, an interview with a young pregnant woman who had tuberculosis during the pandemic in Cape Town, South Africa, showed how systematic flaws in the government’s social support system prevented her from receiving necessary assistance.^19^ Despite her being qualified for multiple support mechanisms, she received only 2 months’ worth of food aid because of stock-outs and delays. Her application for a Social Relief of Distress Grant (SRDG) was rejected because she received a child support grant (CSG) for her eldest child, which was sent to a caregiver in another province. Furthermore, she was not notified of or granted a disability grant despite her dire circumstances, being pregnant and weighing less than 50 kg. This instance demonstrates how social assistance programmes and healthcare providers did not collaborate, leaving her vulnerable and without sufficient financial aid.

### Acceptability

Acceptability refers to the extent to which healthcare services conform to patients’ cultural values, beliefs and standards. It also includes how the attitudes and traits of healthcare practitioners influence patients’ willingness to seek care. The stigma associated with TB significantly affected the acceptability of healthcare services in South Africa before the outbreak. This was especially noticeable in impoverished communities where poverty has been linked to sickness. Patients were frequently subjected to prejudice and social exclusion, which discouraged them from getting treatment quickly.^2,7^ TB-associated stigma was amplified during the COVID-19 pandemic, which further discouraged people from seeking medical attention. Furthermore, healthcare staff faced humiliation and mistrust from their communities because of their role in resolving COVID-19.^7^

## Implications and recommendations

The findings of this scoping review highlight the critical need for structural reforms to address South Africa’s compounded demand-side and supply-side TB care hurdles, particularly in the context of possible public health emergencies. To boost availability, governments should prioritise a steady supply of TB diagnostic devices and medications, ensuring that important services such as TB treatment remain available even during emergencies. Expanding healthcare infrastructure, including telemedicine and mobile healthcare services, can help improve accessibility, especially in rural and underserved areas. Healthcare institutions must also improve accommodation by providing flexible service delivery alternatives and consistent infection control protocols.

To reduce affordability barriers, expanding government social assistance programmes to cover transportation and healthcare costs is critical. Addressing the stigma surrounding TB and COVID-19 through public health campaigns and enhancing healthcare workers’ cultural competence and patient centredness is essential to improving the acceptability of TB services. Implementing these recommendations will strengthen the resilience of the South African health system, ensuring uninterrupted TB care during future health crises.

## Conclusion

All five aspects of access to TB care in South Africa – availability, accessibility, accommodation, affordability and acceptability – have been aggravated by the COVID-19 pandemic. Access to care was hampered by travel restrictions and transportation problems, and availability was impacted by overburdened systems, uneven infection control and limited healthcare resources. Healthcare facilities found it difficult to treat TB patients since they gave priority to COVID-19 cases, and the cost of transportation and lost earnings worsened affordability. Many people felt discouraged from seeking care because of the stigma associated with both COVID-19 and tuberculosis. In order to overcome these obstacles and guarantee that TB services are always available, affordable and accessible – especially in the event of future health emergencies – South Africa has to reinforce its healthcare system, enhance resource management and lessen stigma.
